# Application of Point-of-Care Ultrasound Scanning for the Diagnosis of Perianal Abscess

**DOI:** 10.7759/cureus.21622

**Published:** 2022-01-26

**Authors:** Hiroshi Hori, Hitoshi Sugawara

**Affiliations:** 1 General Medicine, Jichi Medical University Saitama Medical Center, Saitama, JPN

**Keywords:** incision and drainage, perianal abscess, point-of-care ultrasound (pocus), gluteal region, soft tissue infection

## Abstract

A sixty-year-old man with ulcerative colitis and polymyalgia rheumatica, for which he was receiving prednisolone, visited the emergency department complaining of a gradual worsening of pain in the right buttock. Physical examination revealed mild redness and tenderness at the right side of the anus. Ultrasound revealed an approximately 38-mm long, avascular subcutaneous collection with heterogeneous echogenicity in the tender region. A perianal abscess was diagnosed, the lesion was punctured, and pus was drained, after which the pain improved. Point-of-care ultrasonography was effective in the prompt diagnosis and subsequent treatment of this patient’s perianal abscess.

## Introduction

The prevalence of perianal abscess is estimated to be greater than 16.1 per 100,000. Risk factors include inflammatory bowel disease, smoking, human immunodeficiency virus infection, and Crohn's disease [1].

The diagnosis of a perianal abscess is often difficult and time-consuming [1]. Patients usually complain of pain in the pelvis and buttocks but often do not have redness or swelling on visual inspection. Confirmation of pain on rectal examination is important; however, the procedure is very painful for the patient. Perianal abscesses may be associated with sepsis and fistula formation; thus, prompt diagnosis and drainage within 24 hours is recommended [2].

We report the usefulness of point-of-care ultrasonography (POCUS), which can be used as a marker for rapid diagnosis and treatment of perianal abscesses.

## Case presentation

A sixty-year-old man with ulcerative colitis and polymyalgia rheumatica, for which he was routinely taking 20 mg prednisolone and 2000 mg mesalazine, started complaining of pain in the right buttock for five days. The patient had a history of heavy smoking. Because of the gradual worsening of his pain, he visited the emergency department of our hospital.

**Figure 1:**
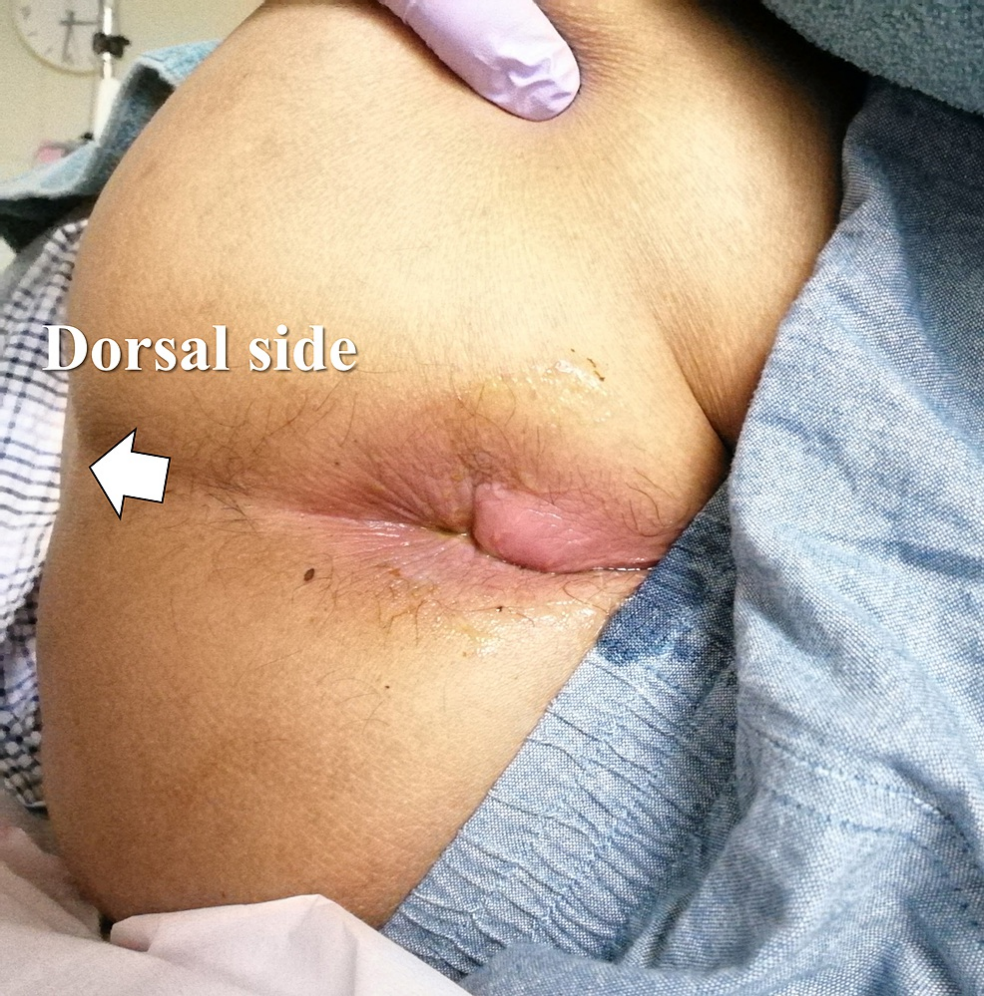
Photograph of buttocks The patient is in the left lateral decubitus position showing redness and mild swelling around the anus. The arrow points to the back and coincides with the direction of the ultrasound body marker.

**Figure 2:**
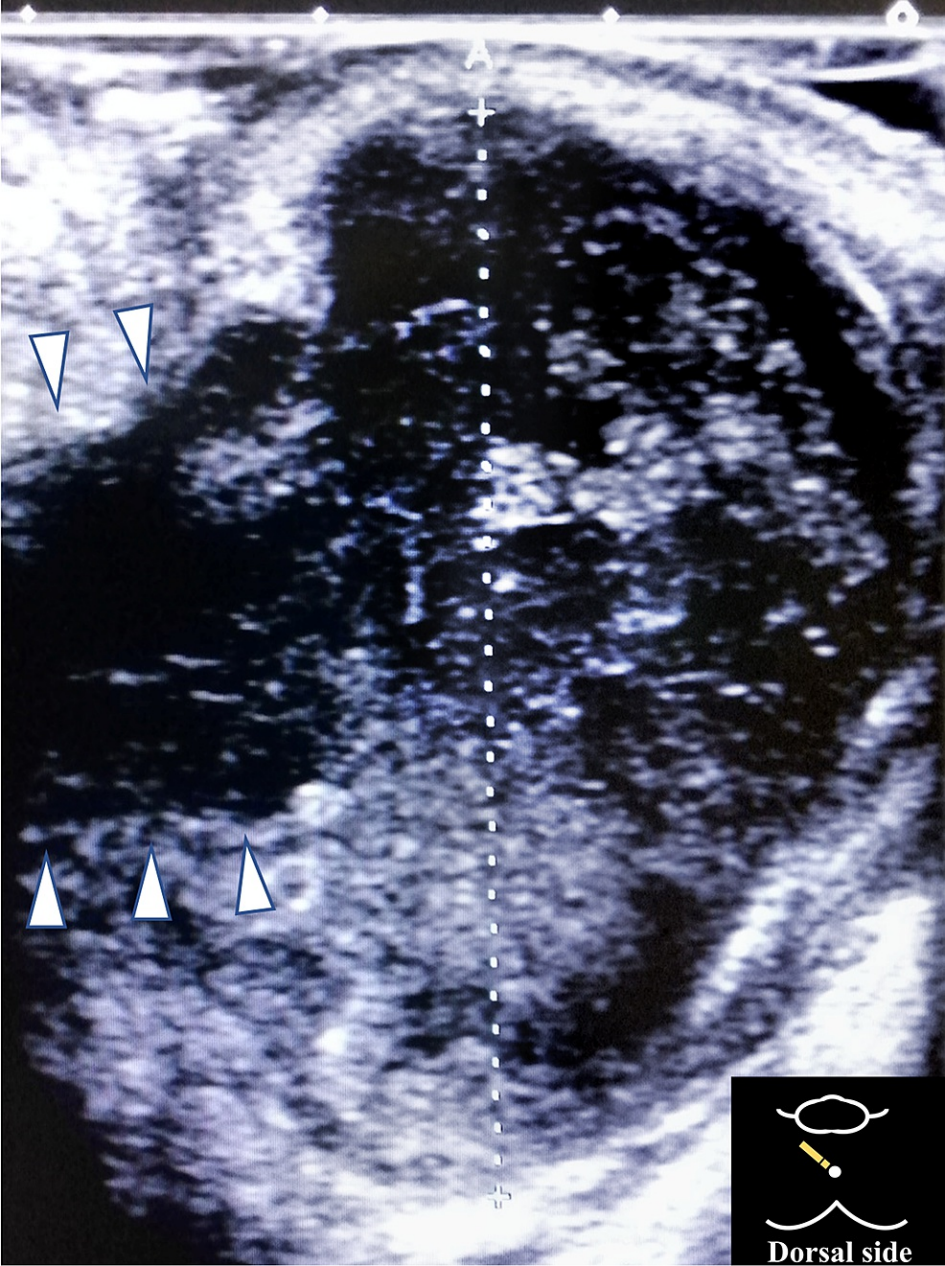
Ultrasound findings The ultrasound probe (linear probe 7.5 Hz) was placed perpendicular to the tender and swollen skin around the anus. It revealed a heteroechoic subcutaneous mass with a hyperechoic rim (diameter 38 mm). Fistulas were observed as hypoechoic tracts (arrowhead) that were continuous with the abscess.

The patient was in a recumbent position and unable to sit because of pain. The patient’s vital signs showed mild tachycardia (heart rate 108/min) and a temperature of 36.7°C. Physical examination revealed mild redness and tenderness around the anus (Figure 1). Differential diagnoses included perianal abscess, cellulitis, thrombosed external hemorrhoids, and malignant tumors. Ultrasound (linear probe 7.5 MHz) revealed a heteroechogenic subcutaneous mass with a hyperechoic rim, measuring 38 mm in diameter, at the site of tenderness, and a perianal abscess was suspected (Figure 2).

The abscess was punctured and drained, and the patient’s pain improved promptly. He was diagnosed with a perianal abscess based on ultrasound findings and drainage of the abscess and was prescribed a daily dose of 1500 mg amoxicillin and 375 mg clavulanic acid for four days. The pain disappeared within four days. POCUS was effective in the prompt diagnosis and treatment of a perianal abscess.

## Discussion

A perianal abscess occurs commonly in immunocompromised patients such as those with diabetes or those taking immunosuppressive medication for autoimmune conditions. Patients with a perianal abscess often lack specific physical examination findings such as erythema and tenderness of perianal skin, which makes it difficult to promptly diagnose and treat. A delayed diagnosis can lead to sepsis and fistula formation. Although perianal abscess is a disease that requires prompt diagnosis and treatment, its diagnosis is time-consuming and costly.

Imaging is the first and most obvious choice of investigation for perianal abscess. Computed tomography (CT) and magnetic resonance imaging (MRI) have limitations in terms of radiation exposure and cost, whereas ultrasound has the advantages of being inexpensive and noninvasive and enabling prompt diagnosis. Compared with ultrasonography, MRI or CT costs approximately three to five times more in Japan. Therefore, our case shows that POCUS can become a popular diagnostic tool, especially in situations where CT may be inaccessible, such as in ambulatory clinics or during home visits.

POCUS includes percutaneous and transanal scanning, and the latter is reported to be effective in diagnosing perianal abscesses [3]; however, it requires an intracavitary probe and technique. In contrast, percutaneous ultrasound scanning with a common linear or convex probe may also be effective for diagnosing a perianal abscess without causing the patient more pain or discomfort. Percutaneous ultrasound has been previously reported to be useful in diagnosing perianal abscesses [4,5].

Using POCUS, a perianal abscess can be diagnosed by following the methods and identifying the features mentioned below [4,6]. A linear (7.5-10 MHz) or a convex probe (3.5-6.0 MHz), with the head of the probe facing the anus, should be placed perpendicular to the tender and swollen skin around the anus. The anal canal should be identified next, which appears as a hypoechoic band (internal sphincter) surrounding the hyperechoic mucosa. A perianal abscess appears as a hypoechoic or heteroechoic mass. Color Doppler imaging does not show blood flow in the abscess. Fistulas are observed as hypoechoic tracts, continuous with the anal canal and abscess, with air bubbles in the lumen. Such ultrasound findings strongly suggest a perianal abscess, and a perianal abscess is diagnosed if pus is drained by puncture. Furthermore, ultrasound-guided drainage is effective. One of its advantages is that it can be easily performed repeatedly, which makes it possible to confirm and completely remove residual abscess using ultrasound-guided drainage [4].

However, POCUS is limited by the difference in reproducibility and subjective difference in the level of ultrasonic procedure acquisition. Some training may be required to perform these diagnostic POCUS and therapeutic procedures accurately. However, it is much simpler than transanal ultrasound, and in this case, the ultrasound procedure was performed by a novice doctor.

## Conclusions

This case shows that POCUS is an effective diagnostic tool for perianal abscess. Therefore, with the widespread availability and cost-effectiveness of POCUS, clinicians should consider using it for the diagnosis and treatment of perianal abscesses.
